# BAG3^+^ CAF-T cell neighborhood predicts resistance to neoadjuvant chemoimmunotherapy in NSCLC

**DOI:** 10.3389/fimmu.2026.1823199

**Published:** 2026-07-07

**Authors:** Jing Sun, Zhengqi Cao, Yueping Jin, Anni Wang, Li Lu, Lixuan Chen, Wenhui Shi, Peiyi Xu, Yuxin Ouyang, Junjie Tang, Zhouwenli Meng, Ziming Li

**Affiliations:** Department of Medical Oncology, Shanghai Chest Hospital, Shanghai Jiaotong University, School of Medicine;Shanghai Key Laboratory of Thoracic Tumor Biotherapy, Shanghai, China

**Keywords:** BAG3^+^ CAF-T cell neighborhood, biomarker, cancer-associated fibroblasts (CAFs), neoadjuvant chemoimmunotherapy (NCIT), non-small cell lung cancer (NSCLC)

## Abstract

**Background:**

Despite significantly improving outcomes in non-small cell lung cancer (NSCLC), neoadjuvant chemoimmunotherapy (NCIT) fails to achieve a major pathological response (MPR) in over 40% of patients. Consequently, the early identification of non-responders prior to treatment initiation remains a critical unmet clinical need.

**Methods:**

In this study, we performed single-cell RNA sequencing (scRNA-seq) on pre-treatment NSCLC tissue samples and integrated data from two public databases to identify signaling pathways associated with poor treatment response. Key findings were subsequently validated using multiplex immunofluorescence (mIF), and the predictive value of identified molecules was finally assessed in our cohort and GEO datasets.

**Results:**

Among the 83 patients, 23/57 (40.35%) of these radiological responders failed to achieve MPR. Data analysis revealed activation of stress-related signaling pathways in cancer-associated fibroblasts (CAFs) and T cells from nMPR patients, with elevated expression of stress-related markers, including BAG3 and IFITM2. MIF confirmed that BAG3^+^IFITM2^+^ CAFs and BAG3^+^CD8^+^ T cells were spatially adjacent and significantly more abundant in nMPR patients. In our cohort and the two public databases, the BAG3^+^ CAF-T Cell Neighborhood was significantly more abundant in the nMPR group compared to the MPR group (*p*<0.05). In the MPR group, there was no significant difference in BAG3^+^ CAF-T Cell Neighborhood between the radiological PR and non-PR subgroups. The AUC values of BAG3^+^IFITM2^+^ CAFs, BAG3^+^CD8^+^ T cells, and the BAG3^+^ CAF-T Cell Neighborhood were 0.84 [95%CI: 0.746-0.931], 0.72 [95%CI: 0.603-0.835], and 0.87 [95%CI: 0.787-0.948], respectively. The OS and DFS of the BAG3^+^ CAF-T Cell Neighborhood high group are significantly decreased than that of the low group (*p*<0.05). The level of BAG3^+^ CAF-T Cell Neighborhood outperformed other two indicators in predicting non-response to NCIT. Consistent results were observed in GSE126044 and GSE135222.

**Conclusion:**

The BAG3^+^ CAF-T Cell Neighborhood may serve as a biomarker for predicting non-response to NCIT in NSCLC, with significant potential to inform clinical decision-making.

## 1 Introduction

Lung cancer remains the malignant tumor with the highest incidence and mortality rate, as non-small cell lung cancer (NSCLC) accounts for approximately 85% (1, 2). Immunotherapy combined with chemotherapy has significantly improved the response rate and survival outcomes of early and advanced stage NSCLC. The CheckMate 816 clinical trials established neoadjuvant chemoimmunotherapy (NCIT) as a new standard of treatment, demonstrating unprecedented pathological complete response (pCR) rates (24% vs 2.2%) and event-free survival (EFS) benefits (31.6 months vs 20.8 months) compared to neoadjuvant chemotherapy alone (3). Thus, NSCLC has entered the era of immune neoadjuvant therapy. The combination of immune checkpoint inhibitors (ICIs) with chemotherapy has demonstrated superior efficacy over chemotherapy alone, achieving significantly improved median EFS (47.2 months vs 18.3months) and 36 months overall survival (OS) (71% vs 64%) in clinical trials KEYNOTE-671 (4). Also, resectable NSCLC patients with tumor PD-L1 levels less than 1% had an EFS benefit with NCIT (5).

Despite its potential, a significant clinical challenge persists: over 40% of patients fail to achieve major pathological response (Pathological-MPR), which correlates with poor survival outcomes. Moreover, NCIT demonstrates limited efficacy, with suboptimal response rates and a subset of patients exhibiting disease progression prior to treatment response, resulting in missed therapeutic opportunities (6). These limitations highlight the urgent need for reliable early predictive biomarkers to identify NCIT non-response patients.

Current biomarkers, such as PD-L1 expression and tumor mutational burden (TMB), exhibit limited sensitivity and specificity in identifying responders of NCIT (7, 8). The OS of advanced NSCLC patients with no response to immunotherapy was only 4.4 months (9). The NADIM II study reinforced the prognostic significance of Pathological-MPR, demonstrating that patients who achieved Pathological-MPR had a significantly higher 2 year OS rate compared to non-responders (10). Radiological assessment via computed tomography (CT) remains the primary modality for evaluating treatment response of NCIT before the surgery. However, emerging evidence highlights a critical discordance between radiological responses and pathological outcomes. For instance, a recent study reported that among patients exhibiting radiological partial response (Radiological-PR) on post-NCIT, 30.23% were subsequently classified as Pathological-nMPR upon surgical resection (11). Thus, it’s important to verify biomarkers that can accurately predict pathological response in order to facilitate personalized treatment adjustments.

Previous study demonstrated that antigen-presenting cancer-associated fibroblasts (apCAFs) induced Treg expansion, contributing to therapeutic resistance (12, 13). CAFs exhibit remarkable heterogeneity (14–16). CAFs have emerged as independent prognostic factors in NSCLC (17–20). These findings collectively underscore the critical role of CAFs in shaping both tumor immunity and treatment response in NSCLC. Our RNA-seq data revealed a novel subset of CAFs: BAG3^+^IFITM2^+^ CAFs were significantly enriched in the TME of non-responders. Bcl2-associated athanogene 3 (BAG3) serves as a critical regulator of cellular homeostasis. Dysregulation of BAG3 has been implicated in oncogenesis, with emerging evidence suggesting its role in tumor progression through modulation of cell cycle regulators and apoptosis-related proteins (21, 22). Interferon induced transmembrane protein 2 (IFITM2) plays a pivotal role in innate immune responses and malignant transformation (23). Genomic analyses have revealed that IFITM2 co-mutates with ANK1 during early clonal evolution in multiple myeloma (24). Notably, studies have identified IFITM2 as a functional receptor for BAG3, with the BAG3-IFITM2 axis demonstrating oncogenic potential. This BAG3-IFITM2 interaction represents a potential therapeutic target, as blockade of this axis significantly reduces tumor growth (25).

TME comprises malignant cells, stromal components, particularly CAFs, and immune cells that collectively influence treatment response (17, 18). Recent studies reveal CAFs can also impair CD8^+^ T cell-induced pyroptosis in tumor cells, further contributing to immune evasion (19). Therefore, CAF, by interacting with other components of the TME, such as CD8^+^ T cells, plays a crucial role in shaping the TME and shows potential value as a prognostic factor and therapeutic target.

We investigated the interaction structure of BAG3^+^IFITM2^+^ CAFs with BAG3^+^CD8^+^ T cells as a novel biomarker for predicting NCIT non-response. Our study aims to validate the BAG3^+^ CAF-T Cell Neighborhood can serve as an effective predictive biomarker, identifying NSCLC patients non-response to NCIT and enable early therapeutic intervention, improving clinical outcomes.

## 2 Materials and methods

### 2.1 Study design and patients

The study enrolled 83 NSCLC patients in Shanghai Chest Hospital between July 2018 and June 2022. The inclusion criteria included: (I) Patients had available pre-treatment tumor samples from primary lung lesions and undergone diagnostic biopsies; (II) Patients confirmed stage II-III NSCLC according to the eighth edition of the tumor, node, metastasis (TNM) classification; (III) Completion of NCIT followed by radical surgery; (IV) Age 18–80 years at diagnosis. Exclusion Criteria: patients with history of other active malignancies and those with incomplete clinical data or loss to follow-up.

The study protocol was approved by the Ethics Committee of Shanghai Chest Hospital (Approval ID: IS25264) and conducted in accordance with the Declaration of Helsinki. Informed consent was obtained from all participants prior to study enrollment.

### 2.2 Efficacy assessment

Radiological assessment by CT: The efficacy assessment of imaging for cancer patients was according to Response Evaluation Criteria in Solid Tumors (RECIST v1.1) guidelines. PR was strictly defined as ≥30% decrease in the sum of longest diameters (SLD) of target lesions compared to baseline measurements and sustained for at least 4 weeks. All cases not meeting these criteria were classified as non-partial response (nPR).

Pathological assessment of surgical specimens: Surgical specimens were evaluated by standardized histopathological protocols. Patients were categorized as follows: pCR: no viable residual tumor cells (0%). MPR: ≤ 10% viable tumor cells remaining, including those with pCR. Non-major pathological response (nMPR): > 10% viable tumor cells.

Survival outcomes: Disease-free survival (DFS): time from surgery to disease recurrence, progression, death from any cause, or last follow-up (whichever occurred first). OS: time from surgery to death from any cause or last confirmed follow-up.

### 2.3 Gene testing and PD-L1 expression tumor proportion score

Genomic alterations were assessed using next-generation sequencing (NGS) platforms. Gene mutation types were recorded. The PD-L1 TPS of the tumor cells was evaluated and categorized into three groups: TPS < 1%, TPS 1–49% and TPS ≥ 50%.

### 2.4 Single-cell RNA sequencing

The scRNA-seq dataset analyzed in this study was derived from our previously generated NCIT NSCLC single-cell cohort. For the present analysis, we re-analyzed the pre-treatment biopsy subset of this cohort, which included 11 NSCLC scRNA-seq libraries. The tissue-processing and library-preparation procedures are briefly summarized below.

Fresh pre-treatment biopsy specimens were transferred in MACS Tissue Storage Solution (Miltenyi Biotec) and kept on ice before dissociation. After brief centrifugation, tumor tissues were incubated with 2 mL of pre-warmed enzymatic digestion solution (Miltenyi Biotec) at 37 °C with gentle pipetting every 5 min. The dissociated cell suspension was filtered through a 40-μm cell strainer, centrifuged at 300 × g for 3 min at 4 °C, treated with ACK lysis buffer to remove red blood cells, and resuspended in PBS containing 0.01% bovine serum albumin.

Single-cell suspensions were loaded into the Chip A Single Cell Kit v2.1 on the MobiNova-100 microfluidic platform (MobiDrop, Zhejiang, China). After droplet encapsulation, barcoded oligonucleotides were released using the MobiNovaSP-100 system. mRNAs were captured by cell barcodes and reverse-transcribed in droplets. Barcoded cDNA libraries were prepared using the High Throughput Single-Cell 3′ Transcriptome Kit v2.1 and 3′ Dual Index Kit (MobiDrop), followed by sequencing on an Illumina NovaSeq 6000 platform.

### 2.5 Publicly available transcriptomic dataset analysis

Public NSCLC immunotherapy transcriptomic datasets were used for external validation. GSE126044 contains bulk RNA-seq data from 16 NSCLC patients treated with anti-PD-1 therapy and annotated as responders or non-responders. GSE135222 contains bulk RNA-seq data from 27 advanced NSCLC patients treated with anti-PD-1/PD-L1 therapy. GSE136961 contains pretreatment tumor expression profiles from 21 NSCLC patients treated with single-agent anti-PD-1 therapy and classified as durable clinical benefit or non-durable benefit according to the original study. GSE93157 contains nCounter PanCancer Immune Profiling Panel data from 65 anti-PD-1-treated patients with melanoma, lung cancer or head and neck cancer; only the NSCLC subset was used in this study. We also analyzed the RNA-seq subset of the SU2C-MARK advanced NSCLC anti-PD-(L)1 cohort reported by Ravi et al. All datasets were analyzed independently.

Expression matrices and clinical annotations were downloaded from GEO or the corresponding publication repository using GEOquery v2.78.0 and R v4.5.2. For RNA-seq datasets, raw or normalized count matrices were converted to gene-level expression matrices, lowly expressed genes were removed, and expression values were log2-transformed after adding a pseudocount when necessary. For processed expression matrices, the authors’ normalized data were used directly after gene-symbol harmonization. For the nCounter dataset, the normalized expression matrix provided by the original study was used. Gene identifiers were converted to official HGNC gene symbols, and duplicated gene symbols were collapsed by retaining the probe or transcript with the highest mean expression across samples. When multiple batches or platforms were present within a dataset, batch effects were adjusted using the removeBatchEffect function in limma v3.66.0. Datasets were not merged for pooled testing.

Cell-state deconvolution was performed using CIBERSORTx with a custom reference built from our annotated scRNA-seq data. To construct the signature matrix, epithelial cells, myeloid cells, endothelial cells, mast cells, B/plasma cells, CAF subclusters and T-cell subclusters were included as separate reference phenotypes. CAF and T-cell states were retained at the subtype level, including BAG3^+^IFITM2^+^ CAFs, other CAF states, BAG3^+^CD8^+^ T cells and other CD8/CD4 T-cell states. The CIBERSORTx “Create Signature Matrix” module was run in single-cell mode using default marker selection settings. The “Impute Cell Fractions” module was then used to estimate cell-state abundance in each bulk or nCounter sample, with 1,000 permutations. Quantile normalization was disabled for RNA-seq and nCounter datasets. S-mode batch correction was applied using the scRNA-seq reference when supported by the CIBERSORTx workflow.

For each public dataset, the CIBERSORTx-inferred abundance of all cell states was extracted. Total CAF abundance was defined as the sum of all CAF-state fractions. The BAG3^+^IFITM2^+^ CAF fraction was defined as the inferred abundance of the BAG3^+^IFITM2^+^ CAF state among all cells, and the BAG3^+^IFITM2^+^ CAF-in-CAF ratio was calculated as the BAG3^+^IFITM2^+^ CAF fraction divided by the sum of all CAF-state fractions. Similarly, total CD8 T-cell abundance was defined as the sum of all CD8 T-cell-state fractions, and the BAG3^+^CD8^+^ T-cell-in-CD8 ratio was calculated as the BAG3^+^CD8^+^ T-cell fraction divided by the sum of all CD8 T-cell-state fractions. A bulk-level BAG3^+^ CAF-T Cell Neighborhood surrogate score was calculated as the mean of the z-scaled log2-transformed CIBERSORTx-inferred BAG3^+^IFITM2^+^ CAF fraction and BAG3^+^CD8^+^ T-cell fraction. Within-lineage ratios were analyzed in parallel to determine whether the observed signal reflected selective enrichment of BAG3-related states rather than only global CAF or CD8 T-cell infiltration.

For response-based analyses, patients were classified according to the original clinical annotations, including responder versus non-responder, durable clinical benefit versus non-durable benefit, early progression versus clinical benefit, or RECIST-defined best overall response when available. Differences in CIBERSORTx-inferred cell-state fractions or surrogate scores between response groups were evaluated using the Wilcoxon rank-sum test. Receiver operating characteristic curves were generated using pROC v1.18.5. Survival analyses were performed using the survival v3.8–3 and survminer v0.5.0 packages. Patients were stratified into high- and low-score groups using the median score within each dataset, and differences in OS or PFS were assessed using the log-rank test. For pathway-level analyses, GSVA v2.4.9 was used to calculate single-sample enrichment scores for MSigDB v2026.1.Hs gene sets, including WANG_RESPONSE_TO_TME_STRESS and REACTOME_ACTIVATION_OF_RESPONSE_TO_STRESS. P values from multi-gene or multi-pathway analyses were adjusted using the Benjamini–Hochberg method.

### 2.6 Single-cell data analysis

Single-cell RNA-seq data processing and analysis.

Raw FASTQ files from the 11 pre-treatment scRNA-seq libraries were processed using Cell Ranger count v6.1.2 (10x Genomics) with the GRCh38 human reference transcriptome. The Cell Ranger-generated filtered feature-barcode matrices were imported into R and analyzed using Seurat v4.4.1. Across the 11 pre-treatment libraries, Cell Ranger initially estimated 105,964 cells before downstream quality-control filtering. Library-level sequencing metrics, including estimated cell number, mean reads per cell, median genes per cell, number of reads and valid barcode rate, are provided in **Supplementary Table 1**.

For downstream analysis, cells were filtered using the same quality-control criteria as in our previous NCIT single-cell analysis. Cells with fewer than 600 or more than 25,000 UMI counts, fewer than 600 detected genes, or more than 5% mitochondrial gene counts were excluded. Ribosomal and hemoglobin-associated gene percentages were also calculated to assess library quality and potential erythrocyte contamination. Ribosomal genes were defined as genes beginning with RPL or RPS, and hemoglobin-associated genes included HBA1, HBA2, HBB, HBD, HBE1, HBG1, HBG2 and HBZ. Potential doublets were identified for each sample independently using DoubletFinder v2.0.4 and removed before integration. Ambient RNA contamination was estimated using decontX v1.4.0, and cells with an estimated contamination score greater than 0.2 were excluded. After UMI/gene/mitochondrial filtering, doublet removal and ambient RNA-based filtering, 81,251 high-quality cells were retained for downstream analyses.

For each sample, raw UMI counts were normalized using the Seurat NormalizeData function with the LogNormalize method and a scale factor of 10,000. The top 2,000 highly variable genes were selected using FindVariableFeatures with selection.method = “vst”. The data were then scaled using ScaleData, with total UMI count and mitochondrial gene percentage regressed out to reduce technical variation. Principal component analysis was performed using RunPCA based on the scaled highly variable genes. To minimize library- and patient-level batch effects, Harmony was applied to the PCA embeddings using sample ID as the batch variable. The Harmony-corrected principal components 1–30 were used for downstream dimensionality reduction, graph construction and clustering.

UMAP visualization was performed using RunUMAP with dimensions 1–30. A shared nearest-neighbor graph was constructed using FindNeighbors with dimensions 1–30, and unsupervised graph-based clustering was performed using FindClusters with a resolution of 0.6. Cluster robustness was evaluated by inspecting canonical lineage markers and by confirming that major clusters were not driven by a single library. Major cell types were annotated according to canonical marker genes, including EPCAM for epithelial cells, COL1A1, COL3A1 and ACTA2 for fibroblasts, CD3D, CD3E, CD4, CD8A and CD8B for T cells, MS4A1 and CD79A for B cells, LYZ, CD68 and LST1 for myeloid cells, PECAM1 and VWF for endothelial cells, TPSAB1 and TPSB2 for mast cells, and MKI67 and TOP2A for proliferating cells. Cell-type annotations were assigned only when clusters showed coherent expression of multiple canonical markers and lacked mutually exclusive lineage markers. Clusters with ambiguous marker expression were re-examined and, where appropriate, excluded from downstream cell-type-specific analyses.

Marker genes for each cluster were identified using the Seurat FindAllMarkers function with the Wilcoxon rank-sum test. Genes were considered cluster-enriched markers if they were detected in at least 25% of cells in the target cluster, had an average log2 fold change greater than 0.25 and showed Bonferroni-adjusted P < 0.05. Differential expression analyses between response groups or between cell states were performed using FindMarkers with the Wilcoxon rank-sum test, min.pct = 0.10 and logfc.threshold = 0.25. Differentially expressed genes were defined as genes with Bonferroni-adjusted P < 0.05 and average log2 fold change > 0.25 unless otherwise specified.

Pathway activities were quantified at the single-cell level using the AddModuleScore function in Seurat. Gene sets related to cellular stress responses, including WANG_RESPONSE_TO_TME_STRESS and REACTOME_ACTIVATION_OF_RESPONSE_TO_STRESS, were obtained from the Molecular Signatures Database. For each gene set, module scores were calculated as the average expression of the genes in the program after subtracting the aggregated expression of randomly selected control genes matched for expression level. Pathway scores were compared between groups using the Wilcoxon rank-sum test, and P values were adjusted for multiple testing using the Benjamini–Hochberg method. For visualization, pathway scores were displayed on UMAP plots, violin plots and heatmaps after scaling within the analyzed cell population.

### 2.7 Multiplexed immunofluorescence assay

Fresh tumor tissues were fixed in 4% polyformaldehyde for more than 24h and then stored in 70% ethanol at 4 °C until processed into paraffin blocks. After the procedures of deparaffinization, rehydration, antigen retrieval, PBS with 3% BSA was added to the tissues to block non-specific binding. Following blocking, tissue sections were incubated with primary antibodies at 4 °C overnight. Signal detection was achieved using horseradish peroxidase (HRP)-conjugated secondary antibodies with tyramide signal amplification (TSA) through a multiple fluorescent immunohistochemical staining kit (Absin, Shanghai, China). Following each tyramide signal amplification (TSA) cycle, slides underwent microwave-mediated antigen retrieval to enhance epitope exposure. Nuclear counterstaining was performed using DAPI after completion of all target antigen labeling. Primary antibodies targeted BAG3 (Proteintech, 10599-1-AP, 1:400), IFITM2 (Proteintech, 12769-1-AP, 1:100), α-SMA (Abcam, ab7817, 1:5000) and CD8 (ZSGB, ZA-0508). To obtain multispectral images, the stained slides were scanned using the PerkinElmer Vectra 3 system (PerkinElmer).

### 2.8 Manual annotation and counting of CAFs and CD8^+^ T cells

The quantities of cell populations were expressed as the number of stained cells per view and as the percentage of positively stained cells among all nucleated cells. Only marker-positive cells were included in analysis. Digital images of the mIF-stained slides were captured at 20x magnification by CaseViewer. The fluorescence intensity thresholds for identifying marker-positive cells were determined individually for each channel based on specific isotype controls and background signal subtraction. Manual annotation and quantification of BAG3, IFITM2, and α-SAM positive cells, as well as BAG3^+^CD8^+^ T cells, were independently performed by two experienced pathologists blinded to all clinical and outcome data. Inter-observer reproducibility was assessed using the intraclass correlation coefficient (ICC) for continuous measurements and Cohen’s kappa coefficient for categorical classifications.

Duplets Neighborhood Abundance was quantified as the number of BAG3^+^IFITM2^+^ CAFs within a 50 μm radius centered on each BAG3^+^CD8^+^ T cell. The 50 μm distance threshold was strictly pre-specified *a priori* based on biological constraints and established landmarks in spatial stroma-immune biology (26–29).

To evaluate the predictive performance of Duplets Neighborhood Abundance for non-MPR status, Receiver Operating Characteristic (ROC) curve analysis was performed. The dataset (N = 73) was randomly split into a training set (70%, n=51) and a validation set (30%, n=22). The optimal cut-off value was determined by maximizing the Youden’s J statistic in the training set and subsequently applied to the validation set. Area Under the Curve (AUC) was calculated to assess discriminative ability. The 95% Confidence Intervals (CIs) for AUC were estimated using a bootstrapping method with 2000 resamples. Sensitivity and specificity were calculated at the established threshold. Duplets Neighborhood Abundance using a cut-off of ≥2. In addition, 2 corresponded to the median value. BAG3^+^IFITM2^+^ CAFs proportion using the median value (10%) as the cut-off.

### 2.9 Western blotting

Total protein was extracted using RIPA lysis buffer supplemented with protease and phosphatase inhibitors. Protein concentrations were determined using a BCA Protein Assay Kit. Equal amounts of protein were separated by SDS-PAGE, transferred onto PVDF membranes, and incubated with primary (Proteintech, BAG3) (Proteintech, IFITM2) and HRP-conjugated secondary antibodies. Protein bands were visualized using an enhanced chemiluminescence (ECL) detection system.

### 2.10 RNA extraction and RT-qPCR

Total RNA was extracted using TRIzol reagent and reverse-transcribed into cDNA. Quantitative real-time PCR was performed using SYBR Premix Ex Taq on a LightCycler 480 System. Relative gene expression was calculated using the 2^−ΔΔCt method with GAPDH as the internal control.

### 2.11 siRNA transfection

Specific siRNAs targeting BAG3 or IFITM2 and a scrambled negative control siRNA were synthesized by Obio Technology (Shanghai, China). Cells were transfected with siRNAs using Lipofectamine 3000 according to the manufacturer’s instructions. Knockdown efficiency was evaluated by RT-qPCR and western blotting.

### 2.12 Transwell co-culture assay

Fibroblasts were seeded in the upper chamber of a 0.4-μm transwell system, while human PBMC-derived or mouse splenic CD8^+^ T cells were placed in the lower chamber. Cells were co-cultured for 48 h under standard culture conditions, after which CD8^+^ T cells were collected for further analysis.

### 2.13 Flow cytometry

Following co-culture, CD8^+^ T cells were harvested and stained with fluorochrome-conjugated antibodies against live/dead, CD45, CD8, CD3, IFN-γ and Granzyme B (GZMB). Data were acquired using a FACSVerse flow cytometer (BD Biosciences) and analyzed with FlowJo software (10.8.1). The proportions of IFN-γ^+^ CD8^+^ T cells and GZMB^+^ CD8^+^ T cells were quantified.

### 2.14 Statistical analysis

Statistical analyses were conducted using SPSS version 25.0 (IBM Corp., Armonk, NY, USA) and GraphPad Prism version 9.0 (GraphPad Software, San Diego, CA, USA). Data were analyzed using R. Data are presented as mean ± SEM or SD, as indicated. One-way ANOVA, Pearson χ2 or Fisher’s exact test were used to compare the baseline characteristics of groups. Student’s t-test was applied to compare the number of CAFs of samples obtained from groups. The Kaplan-Meier method was employed to estimate DFS and OS. The receiver operating characteristic curve (ROC) was used to evaluate the predictive value. *P*<0.05 was considered statistically significant.

## 3 Results

### 3.1 Patient characteristics

This study included 83 NSCLC patients. Baseline and clinical characteristics are summarized in **Table 1**. The median age was 61 years, with a male predominance (86.75%). Pre-treatment clinical stage distribution was: stage II (14.46%), stage III (85.54%). Postoperative pathological stage: pCR (18.07%), I (25.30%), II (18.07%), and III (38.55%). MPR was achieved in 50.60% of patients, while 49.40% showed nMPR. Histologically, squamous cell carcinoma (62.65%) was most common, followed by adenocarcinoma (28.92%) and other subtypes (8.43%). The cohort demonstrated the following PD-L1 TPS distribution: PD-L1 <1%: 26.51%; PD-L1 1–49%: 21.69%; PD-L1 ≥50%: 27.71%; undetected: 24.1%%.

**Table 1:** Characteristics of all patients.

Characteristics	Patients (n=83)
Age, years [median]	61
Gender (n, %)	
Male	72 (86.75%)
Female	11 (13.25%)
Clinical stage(n, %)	
II	12 (14.46%)
III	71 (85.54%)
yp stage(n, %)	
pCR	15 (18.07%)
I	21 (25.30%)
II	15 (18.07%)
III	32 (38.55%)
Pathological Response(n, %)	
MPR	42 (50.60%)
nMPR	41 (49.40%)
Histologic subtype(n, %)	
Adenocarcinoma	24 (28.92%)
Squamous cell carcinoma	52 (62.65%)
other	7 (8.43%)
PD-L1 expression(n, %)	
< 1%	22 (26.51%)
1–50%	18 (21.69%)
≥ 50%	23 (27.71%)
Unknown	20 (24.1%)

pCR, pathological complete response; MPR, major pathological response; nMPR, non-MPR.

**Figure 1:**
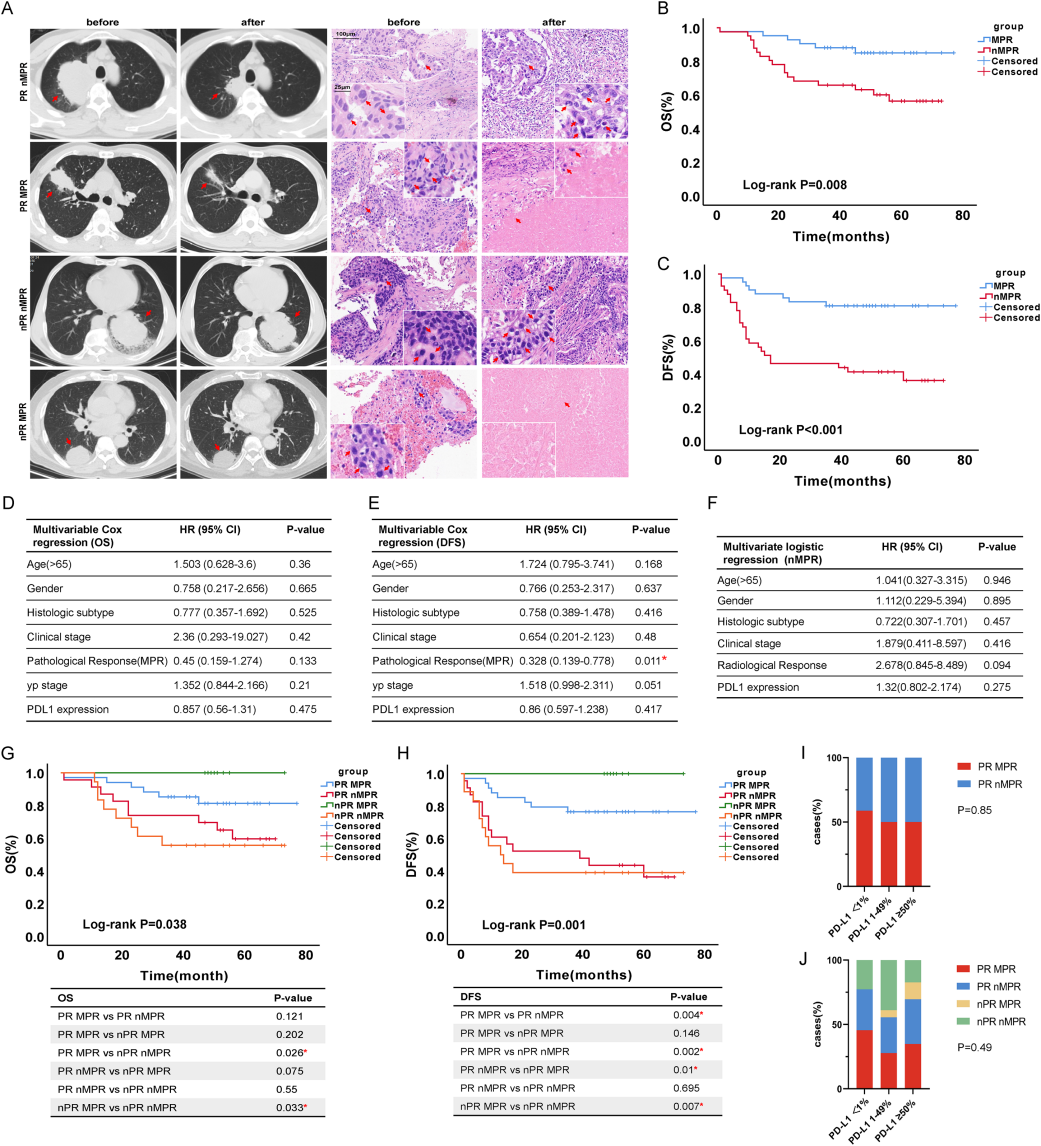
Patient grouping and analysis. **(A)** Radiological images of NSCLC patients from 4 groups before and after NCIT. The red arrow indicates a typical tumor lesion (left). Pathological diagnosis images of NSCLC from 4 groups before and after NCIT. The red arrow indicates typical tumor cells. Scale bar, 100 μm (right). **(B)** Kaplan–Meier curves analysis of OS. **(C)** Kaplan-Meier curves analysis of DFS. **(D)** Multivariable Cox regression for OS. **(E)** Multivariable Cox regression for DFS. **(F)** Multivariable logistic regression for nMPR. **(G)** Kaplan–Meier curves analysis of OS among the 4 groups. **(H)** Kaplan–Meier curves analysis of DFS among the 4 groups. **(I)** The proportions of different PD-L1 expression levels in the 2 groups. **(J)** The proportions of different PD-L1 expression levels among the 4 groups.

Our analysis revealed a clinically significant discordance between radiological and pathologic response assessments. Notably, 40.35% PR patients failed to achieve MPR. Based on this observation, we stratified patients into four distinct response categories: PR-MPR, PR-nMPR, nPR-MPR, nPR-nMPR (**Figure 1A**). A comprehensive comparison of baseline clinical characteristics across these four groups is presented in **Table 2**. No significant differences were observed in baseline characteristics except ypStage (*p*<0.001). Patients with MPR demonstrated significantly improved survival outcomes compared to nMPR cases. MPR group showed significantly increased OS (*p*=0.008) (**Figure 1B**). DFS in nMPR group was significantly shorter than that in MPR group (*p*<0.001) (**Figure 1C**). Multivariable Cox regression analysis for OS demonstrated that none of the seven variables included in the model was significantly associated with OS (**Figure 1D**). Multivariate Cox regression analysis identified MPR status as a prognostic factor for DFS (HR 0.328, 95% CI: 0.139-0.778) (*p*=0.011) (**Figure 1E**). Multivariable logistic regression analysis for nMPR demonstrated none of the variables included in the model was significantly associated with nMPR (**Figure 1F**). Significant differences of OS observed across all four response groups (*p*=0.038) (**Figure 1G**). PR-MPR group showed numerically superior OS compared to PR-nMPR group (*p*=0.121). DFS showed marked differences among groups (*p*=0.001) (**Figure 1H**). PR-MPR patients demonstrated significantly prolonged DFS vs PR-nMPR (*p*=0.004).

**Table 2:** Characteristics of patients in different 4 groups. .

Characteristics	PR-MPR [n =34]	PR-nMPR [n =23]	nPR-MPR [n =8]	nPR-nMPR [n =18]	*P*-value
Age, years [median]	62	61	63	60	0.8
Gender (n, %)					0.84
Male	29 (85.29%)	20 (86.96%)	8 (100%)	15 (83.33%)	
Female	5 (14.71%)	3 (13.04%)	0	3 (16.67%)	
Clinical stage(n, %)					0.61
II	7 (20.6%)	3 (13%)	0	2 (11.1%)	
III	27 (79.4%)	20 (87%)	8 (100%)	16 (88.9%)	
yp stage(n, %)					<0.001*
pCR	14 (41.18%)	0	1 (12.50%)	0	
I	9 (26.47%)	7 (30.43%)	3 (37.50%)	2 (11.11%)	
II	5 (14.71%)	3 (13.04%)	2 (25.0%)	5 (27.78%)	
III	6 (17.65%)	13 (56.52%)	2 (25.0%)	11 (61.11%)	
Histologic subtype(n, %)					0.33
Squamous cell carcinoma	22 (64.71%)	13 (56.52%)	6 (75.0%)	11 (61.11%)	
Adenocarcinoma	7 (20.59%)	10 (43.48%)	2 (25.0%)	5 (27.78%)	
other	5 (14.71%)	0	0	2 (11.11%)	
PD-L1 expression(n, %)					0.23
< 1%	10 (29.41%)	7 (30.43%)	0	5 (27.78%)	
1–50%	5 (14.71%)	5 (21.74%)	1 (12.50%)	7 (38.89%)	
≥ 50%	8 (23.53%)	8 (34.78%)	3 (37.50%)	4 (22.22%)	
Unknown	11 (32.35%)	3 (13.04%)	4 (50.0%)	2 (11.11%)	

Radiological response: PR, partial response; nPR, non-PR. Pathological response: MPR, major pathological response; nMPR, non-MPR; pCR, pathological complete response.

### 3.2 Gene testing: mutation profiles in groups

NGS revealed comparable mutation frequencies for key driver genes (EGFR, KRAS, ALK, TP53, PI3KCA, CDKN2A) between groups. No significant differences in mutation rates for any analyzed gene between the PR-MPR group and the PR-nMPR group (*p*>0.05) (**Supplementary Figure 1A**). There was no difference in mutation rates among the four groups (*p*>0.05) (**Supplementary Figure 1B**). Wild-type (non-mutated) cases similarly showed no intergroup differences (*p*>0.05) (**Supplementary Figures 1C, D**).

### 3.3 PD-L1 expression and survival outcomes

A trend toward improved survival was observed in PD-L1-high (PD-L1≥50%) patients for both OS and DFS, but these differences did not reach statistical significance (*p*>0.05) (**Supplementary Figures 1E, F**). In the PR-MPR and PR-nMPR groups, there was no significant statistical difference in the expression level of PD-L1 (*p*=0.85) (**Figure 1I**). The comparisons among the four groups also yielded the same conclusion (*p*=0.49) (**Figure 1J**). Our findings indicated that PD-L1 expression lacked sufficient prognostic value.

### 3.4 Enrichment of stress-related pathways and expansion of BAG3^+^IFITM2^+^CAFs in non-responders

**Figure 2:**
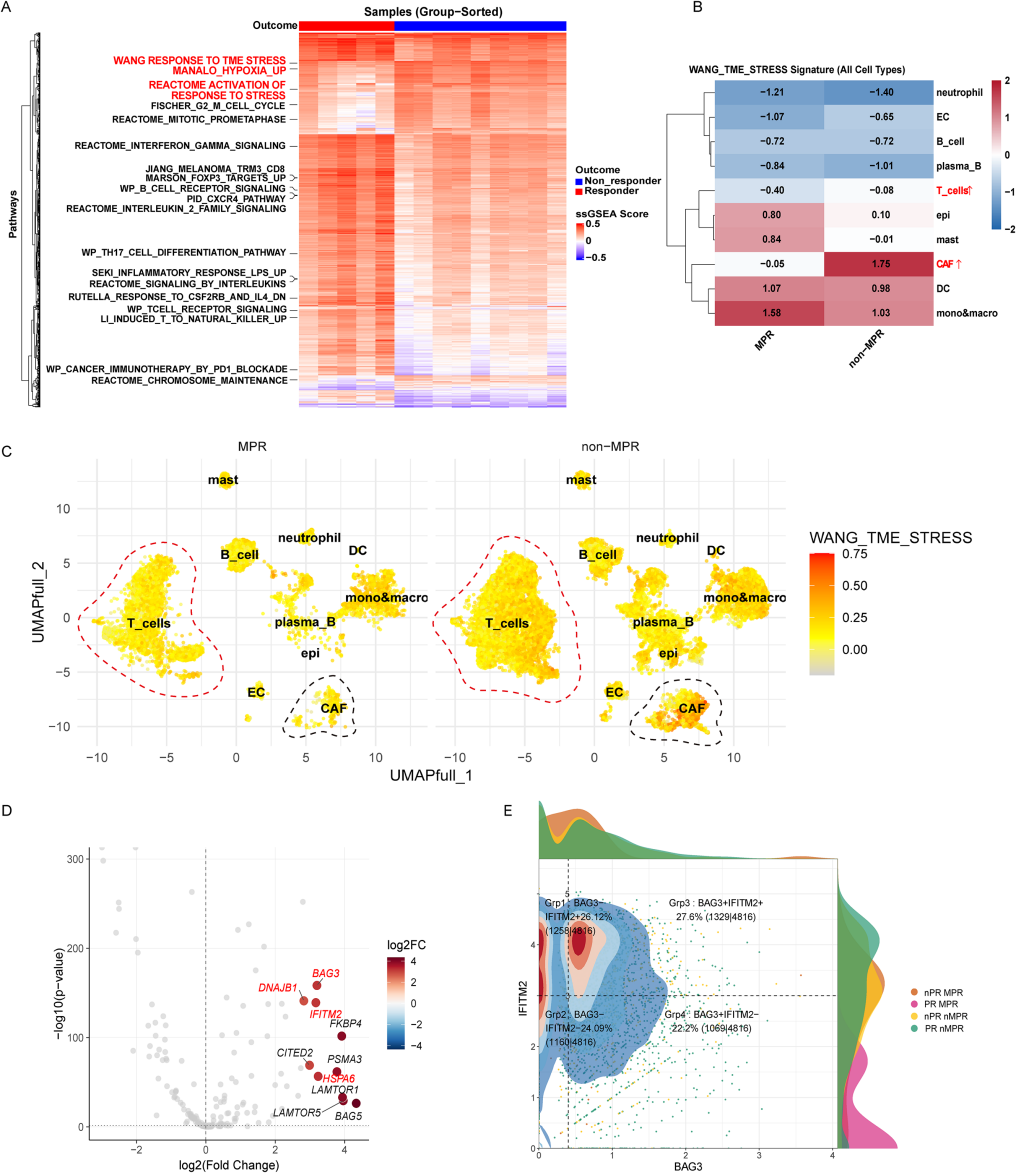
Activated pathways and molecules. **(A)** In public databases GSE126044+GSE135222, significantly activated pathways in non-response patients to ICI treatment than in responders. **(B)** The heatmap showed RESPONSE TO TME STRESS pathways in CD8^+^ T cells and CAFs were higher in nMPR than the MPR cases. **(C)** UMAP plot showing the expression of WANG TME STRESS pathways in CD8^+^ T cells and CAFs were higher in nMPR patients. **(D)** Volcano plot showing abnormally enriched markers in nMPR group. **(E)** Flow cytometric scatterplots showing the number of BAG3^+^IFITM2^+^ CAFs is higher in the PR-nMPR group than other groups.

The analysis results from the GSE126044 and GSE135222 datasets showed that stress-related pathways in No Response (NR) patients were significantly activated, especially the pathways WANG RESPONSE TO TME STRESS and REACTOME ACTIVATION OF RESPONSE TO STRESS (**Figure 2A**), compared with responders. The heatmap showed that the cells with abnormal activation of WANG RESPONSE TO TME STRESS in the nMPR groups were CAFs and T cells (**Figure 2B**). The cluster map showed that the cells with significantly upregulated expression of WANG RESPONSE TO TME STRESS were CAFs and T cells in nMPR group, compared with MPR group (**Figure 2C**). Then the volcano plot indicated that a large number of stress-related markers were upregulated in nMPR group, including BAG3 and IFITM2 (**Figure 2D**). The analysis showed that BAG3^+^IFITM2^+^ CAFs were significantly enriched in the PR-nMPR group compared with other groups (**Figure 2E**).

### 3.5 BAG3^+^IFITM2^+^ CAFs negatively correlated with survival

**Figure 3:**
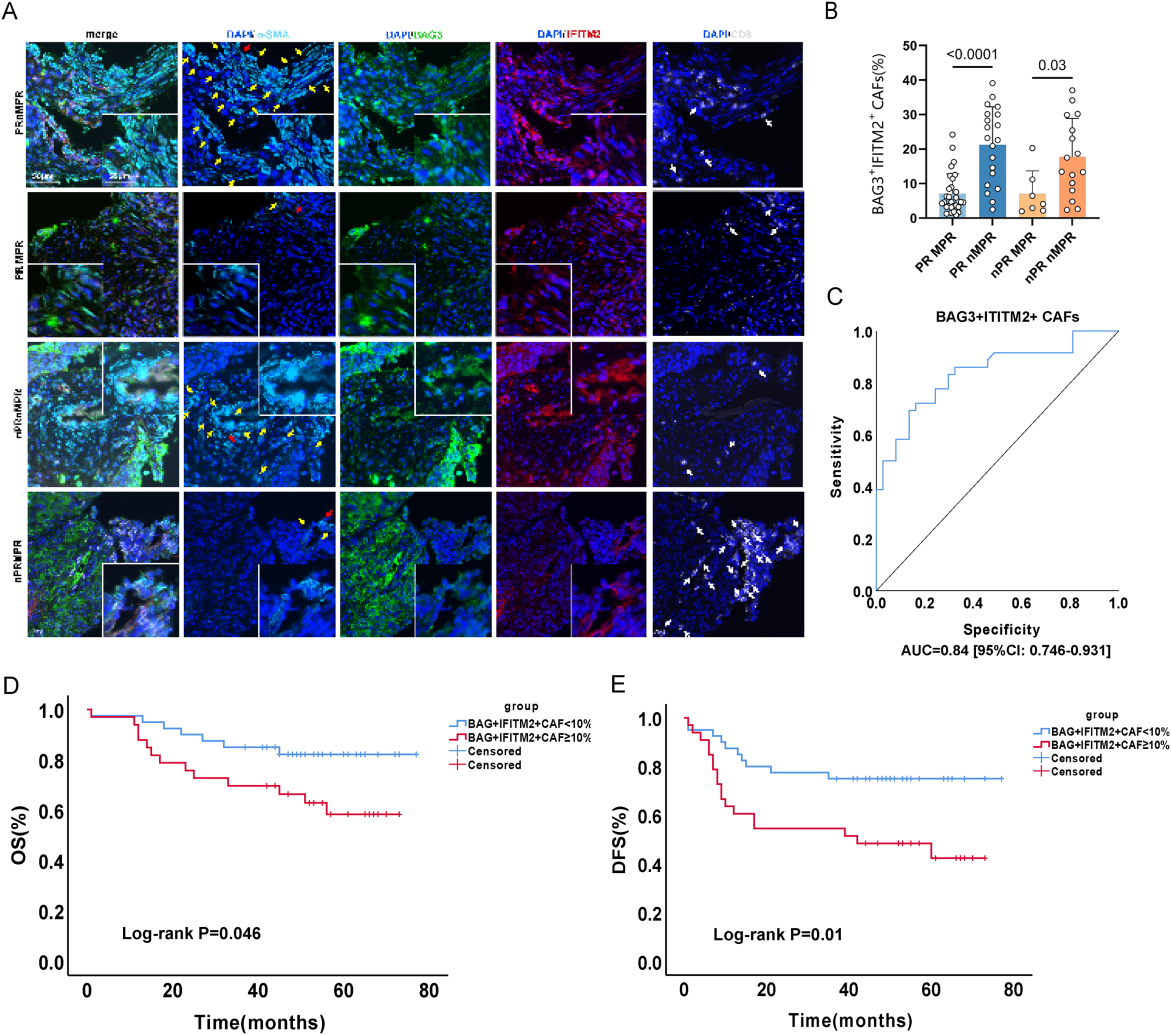
The analysis of mIF. **(A)** The mIF showed the distributions of BAG3^+^IFITM2^+^ CAFs and CD8^+^ T cells in the 4 groups (n=20, 30, 16, 7). The yellow arrows indicate the spatial distribution of BAG3^+^IFITM2^+^ CAFs. The red arrow indicates the enlarged area. The white arrows indicate the spatial distribution of CD8^+^ T cells. **(B)** Bar chart showing the proportion of BAG3^+^IFITM2^+^ CAFs in each group. **(C)** ROC curve of BAG3^+^IFITM2^+^ CAFs; AUC and 95% CI. **(D)** Kaplan–Meier curves analysis of OS between the BAG3^+^IFITM2^+^ CAFs high and low groups. **(E)** Kaplan–Meier curves analysis of DFS between the BAG3^+^IFITM2^+^ CAFs high and low groups.

Subsequently, we detected the expression of BAG3, IFITM2, α-SAM and CD8 in NSCLC tissue samples before NCIT by mIF (n=83). We initially observed a subset of CAFs within TME exhibiting co-localization of BAG3, IFITM2 and α-SAM, confirming the presence of BAG3^+^IFITM2^+^ CAFs (**Figure 3A**). The mIF analysis results showed a significant increase in the level of BAG3^+^IFITM2^+^ CAFs in the nMPR patients, PR-MPR vs PR-nMPR (*p*<0.0001) and nPR-MPR vs nPR-nMPR (*p*=0.03) (**Figure 3B**). However, the proportion of CD8^+^ T cells showed no significant difference except nPR-MPR and nPR-nMPR groups (**Supplementary Figure 1G**). We generated the ROC curve of BAG3^+^IFITM2^+^ CAFs, with an area under the curve (AUC) of 0.84 [95%CI: 0.746-0.931] (**Figure 3C**). We defined BAG3^+^IFITM2^+^ CAFs as ‘high’ when their proportion was≥10% and ‘low’ when it was<10%, followed by performing Kaplan-Meier (KM) survival analysis. In the PR-nMPR group, the case of patients with high BAG3^+^IFITM2^+^ CAFs was significantly higher than that in the PR-MPR group (**Supplementary Figure 1H**). Similarly, the number of patients with high CAFs was significantly higher in the nPR-nMPR group than in the nPR-MPR group (**Supplementary Figure 1I**). There were significant differences in the distribution of CAFs among the four groups (**Supplementary Figure 1J**). The results showed that patients in the high BAG3^+^IFITM2^+^ CAFs group had significantly decreased OS (*p*=0.046) (**Figure 3D**), and significantly shorter DFS (*p*=0.01) (**Figure 3E**). These results confirm that the level of pre-treatment BAG3^+^IFITM2^+^ CAFs correlates with NCIT efficacy and can effectively predict non-response to NCIT treatment.

### 3.6 Spatial proximity and cellular interactions between BAG3^+^IFITM2^+^ CAFs and CD8^+^ T cells

**Figure 4:**
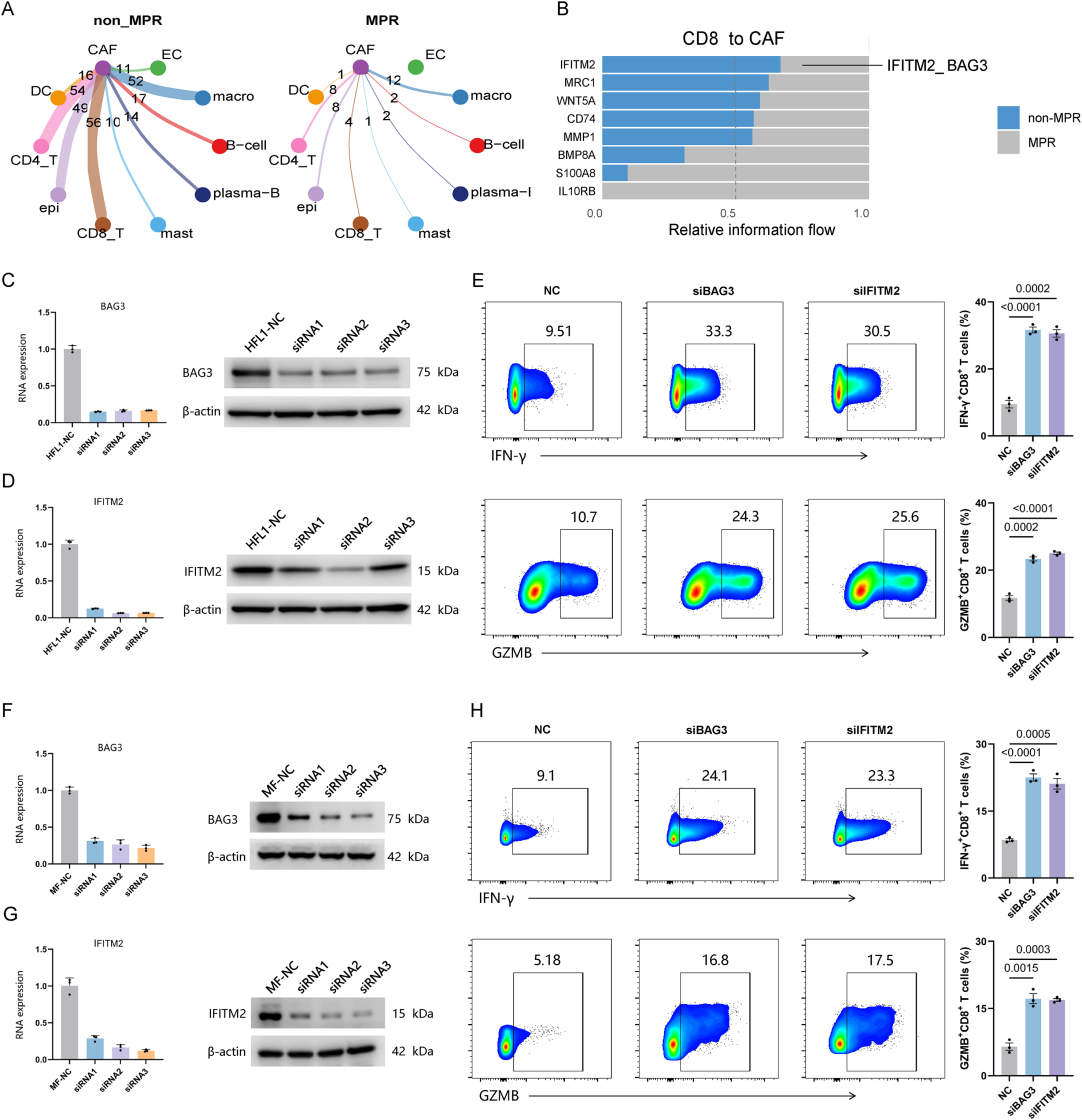
BAG3^+^IFITM2^+^ CAFs interacted with CD8^+^ T cells. **(A)** CellChat plot analysis revealed the interaction between CAFs and other cells. **(B)** Ranked bar plot showing that CD8^+^ T cells interact with CAFs via BAG3-IFITM2 axis. **(C, D)** qPCR and Western blot analyses confirmed the successful siRNA-mediated knockdown of BAG3 and IFITM2 in HFL1 cells. **(E)** Representative flow cytometry plots and quantification of IFN-γ^+^CD8^+^ T cells and GZMB^+^ CD8^+^ T cells among human CD8^+^ T cells. **(F, G)** qPCR and Western blot analyses confirmed the successful siRNA-mediated knockdown of BAG3 and IFITM2 in MF cells. **(H)** Representative flow cytometry plots and quantification of IFN-γ^+^CD8^+^ T cells and GZMB^+^CD8^+^ T cells among mouse CD8^+^ T cells.

Bioinformatics analysis results showed that CD8^+^ T cells are the cells with the strongest interaction with CAFs besides CD4^+^ T cells in the Pathological-nMPR patients (**Figure 4A**). Interaction pair screening revealed that CD8^+^ T cells interact with CAFs through the BAG3-IFITM2 axis (**Figure 4B**). To elucidate the role of the BAG3–IFITM2 axis in regulating CD8^+^ T-cell function, BAG3 and IFITM2 were individually silenced in human fibroblasts (HFL1 cells) using siRNA. Successful knockdown was confirmed by quantitative PCR and Western blot analyses (**Figures 4C, D**). HFL1 cells with BAG3 or IFITM2 knockdown were subsequently co-cultured with CD8^+^ T cells isolated from peripheral blood mononuclear cells (PBMCs). Flow cytometric analysis demonstrated a significant increase in the proportion of IFN-γ^+^CD8^+^ T cells and GZMB^+^CD8^+^ T cells in the BAG3 and IFITM2-knockdown groups compared with the control group (**Figure 4E**). Consistent with the results obtained in human HFL1 fibroblasts, BAG3 and IFITM2 were individually silenced in mouse fibroblasts (MF) using siRNA (**Figures 4F, G**). The knockdown fibroblasts were then co-cultured with CD8^+^ T cells isolated from murine splenic mononuclear cells (SMC). Flow cytometric analysis demonstrated significantly increased proportions of IFN-γ^+^CD8^+^ T cells and GZMB^+^CD8^+^ T cells in the BAG3 and IFITM2-knockdown groups compared with the control group (**Figure 4H**), indicating enhanced CD8 T-cell activation and cytotoxic function. Collectively, these results suggest that the BAG3-IFITM2 axis exerts an inhibitory effect on CD8^+^ T-cell function.

**Figure 5:**
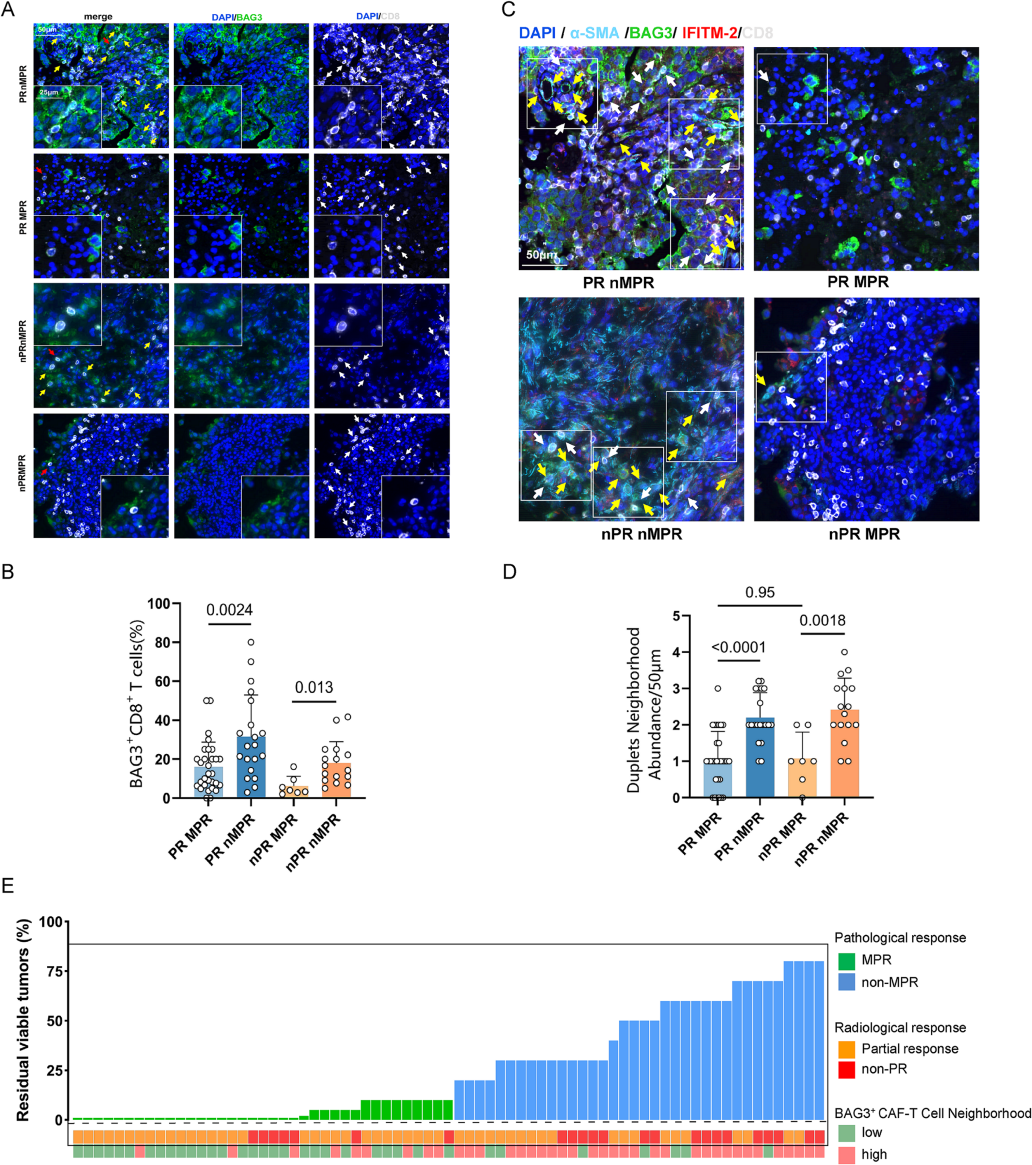
BAG3^+^IFITM2^+^ CAFs and BAG3^+^CD8^+^ T cells constitute the duplets neighborhood. **(A)** mIF showing the distribution of BAG3^+^CD8^+^ T cells in 4 groups (n=20, 30, 16, 7). The yellow arrows indicate BAG3^+^CD8^+^ T cells. The red arrow indicates the enlarged area. The white arrows indicate the CD8^+^ T cells. **(B)** Bar chart showing the proportion of BAG3^+^CD8^+^ T cells in 4 groups. **(C)** The Duplets Neighborhood consisted of BAG3^+^IFITM2^+^ CAFs and BAG3^+^CD8^+^ T cells (n=20, 30, 16, 7). The yellow arrows indicate BAG3^+^IFITM2^+^ CAFs. The white arrows indicate BAG3^+^CD8^+^ T cells. **(D)** Bar chart showing the number of Duplets Neighborhood in 4 groups. **(E)** Waterfall chart illustrating proportion of residual viable tumors in patients, as assessed by pathological examination of surgically resected tumor samples. The dashed line represents the cutoff for major pathological response (10% residual viable tumors). The radiological response and the level of BAG3^+^ CAF-T Cell Neighborhood of each patient was also evaluated and color-coded beneath the chart.

Also mIF confirmed the existence of BAG3^+^CD8^+^ T cells (**Figure 5A**), and further analysis of their infiltration revealed that the infiltration level of BAG3^+^CD8^+^ T cells was significantly increased in the PR-nMPR (*p*=0.0024) and nPR-nMPR (*p*=0.013) groups (**Figure 5B**). In the mIF, BAG3^+^IFITM2^+^ CAFs and BAG3^+^CD8^+^ T cells are spatially adjacent (**Supplementary Figure 2A**).

### 3.7 The level of BAG3^+^ CAF-T Cell Neighborhood positively associated with non-response to NCIT

We defined the spatial adjacency and interaction between BAG3^+^IFITM2^+^ CAFs and BAG3^+^CD8^+^ T cells as the ‘BAG3^+^ CAF-T Cell Neighborhood’. The calculation method of the BAG3^+^ CAF-T Cell Neighborhood is the number of BAG3^+^IFITM2^+^ CAFs within a 50-μm radius centered on BAG3^+^CD8^+^ T cell. The mIF results showed the levels of the BAG3^+^ CAF-T Cell Neighborhood were significantly increased in both the PR-nMPR (*p*<0.0001) and nPR-nMPR (*p*=0.0018) groups (**Figures 5C, D**). In the MPR group, there was no significant difference in BAG3^+^ CAF-T Cell Neighborhood between the radiological PR and non-PR subgroups. Patients were stratified into the ‘high’ group (BAG3^+^ CAF-T Cell Neighborhood ≥ 2) and the ‘low’ group (< 2). Inter-observer reproducibility was excellent, with an ICC of 0.88 [95% CI: 0.821–0.923] for continuous measurements and a Cohen’s kappa coefficient of 0.85 for high/low classification.

The patient-flow diagram and the waterfall chart show that 80.56% (29/36) of patients with nMPR have high levels of BAG3^+^ CAF-T Cell Neighborhood. However, among 36 patients with nMPR, 20 (55.56%) cases were radiological PR (**Figure 5E**, **Supplementary Figure 2B**). These results indicate that the level of the BAG3^+^ CAF-T Cell Neighborhood is positively associated with nMPR for NCIT treatment.

### 3.8 BAG3^+^ CAF-T Cell Neighborhood in TME as a predictive biomarker for NCIT non-response in NSCLC

The ROC curve of BAG3^+^CD8^+^ T cells was generated, with an AUC of 0.72 [95%CI: 0.603-0.835] (**Supplementary Figure 2C**), indicating that its predictive performance was inferior to that of BAG3^+^IFITM2^+^ CAFs.

**Figure 6:**
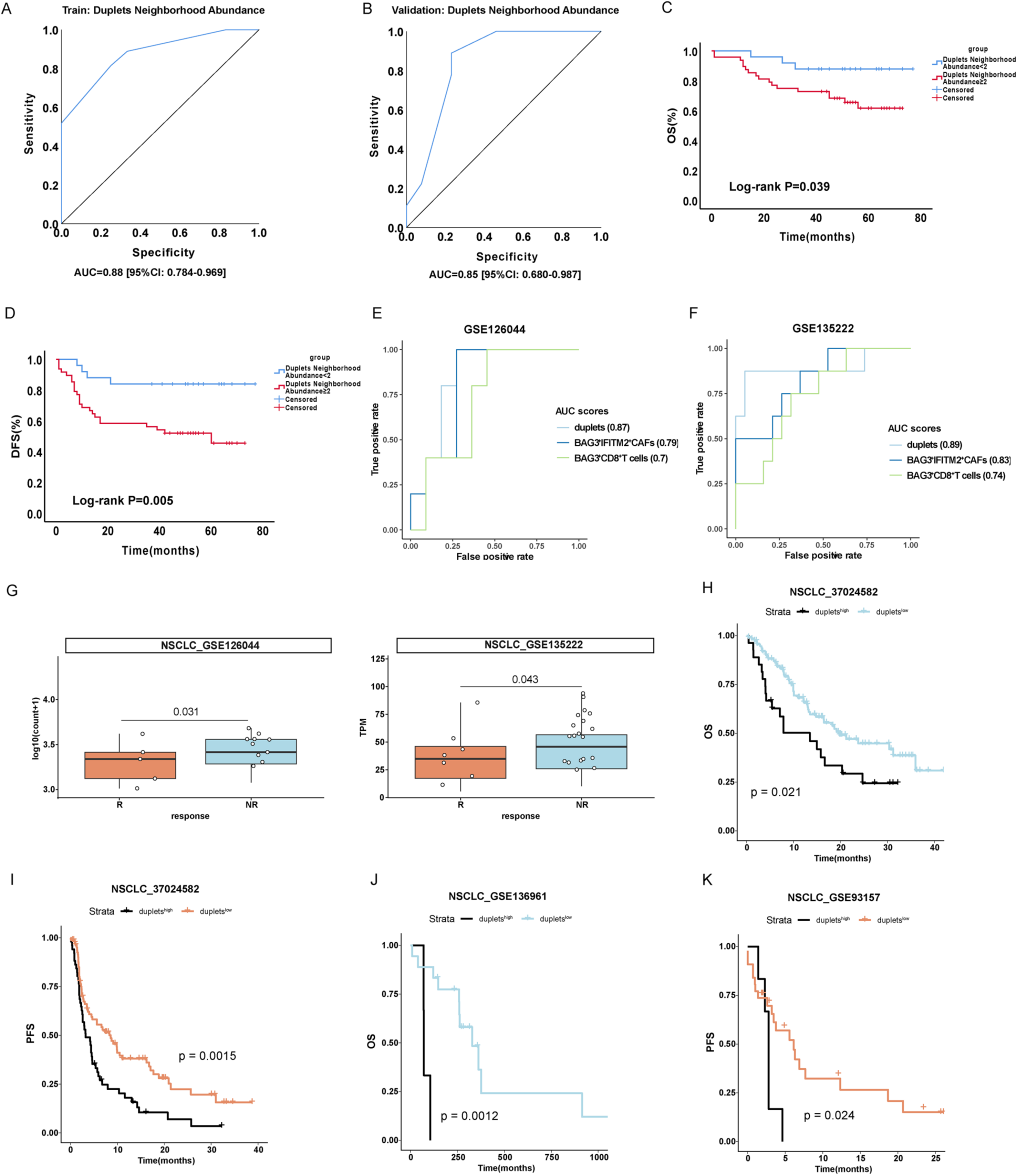
BAG3^+^ CAF-T cell neighborhood predicts NCIT non-response. **(A)** ROC curve for the training set (n=51); AUC and 95% CI. **(B)** ROC curve for the validation set (n=22) applying the pre-defined cut-off value of 2.0; AUC and 95% CI. **(C)** Kaplan–Meier curves analysis of OS between the BAG3^+^ CAF-T cell neighborhood high and low groups. **(D)** Kaplan–Meier curves analysis of DFS between the BAG3^+^ CAF-T cell neighborhood high and low groups. **(E)** ROC curves of 3 molecules in GSE126044. **(F)** ROC curves of 3 molecules in GSE135222. **(G)** NSCLC-GSE126044: centralized signature score of duplets in non-response (NR) group and response (R) group (left). NSCLC- GSE135222: normalized signature score of duplets in NR group and R group (right). **(H, I)** The survival analysis was performed according to duplets in NSCLC-GSE37024582. **(J)** NSCLC-GSE136961: The OS of the duplets low group is significantly better than the high group. **(K)** NSCLC-GSE93157: The PFS of the duplets high group is significantly decreased.

To determine whether Duplets Neighborhood Abundance could serve as a predictive marker for non-MPR status, we conducted ROC curve analyses. The total cohort consisting of 73 patients was randomly divided into a training cohort (n=51) and a validation cohort (n=22). In the training cohort, Duplets Neighborhood Abundance demonstrated robust predictive capability with an AUC of 0.88 [95%CI: 0.784–0.969] (**Figure 6A**). The optimal cut-off value was determined to be 2.0 based on the maximum Youden index, yielding a sensitivity of 81.48% and a specificity of 75.00%.

To verify these findings, the derived cut-off value of 2.0 was applied to the independent validation cohort. The predictive performance remained consistent, achieving an AUC of 0.85 [95%CI: 0.680–0.987] (**Figure 6B**). At this threshold, the sensitivity and specificity in the validation cohort were 77.78% and 76.92%, respectively. Furthermore, analysis of the entire dataset (N = 73) yielded an overall AUC of 0.87 [95%CI: 0.787-0.948] (**Supplementary Figure 2D**). ROC curve analysis performed in the PR subgroup demonstrated that the BAG3^+^ CAF–T cell neighborhood achieved an AUC of 0.86 [95%CI: 0.756-0.960] (**Supplementary Figure 2E**), suggesting excellent predictive performance even among patients with partial response. These results indicate that Duplets Neighborhood Abundance is a reliable indicator for predicting non-MPR status. Patients with a high level of BAG3+ CAF-T Cell Neighborhood had significantly decreased OS (*p*=0.039) and DFS (*p*=0.005) than those with a low level (**Figures 6C, D**). In GSE126044 database, the AUC scores of duplets, BAG3^+^IFITM2^+^ CAFs and BAG3^+^CD8^+^ T cells were 0.87, 0.79, and 0.7, respectively (**Figure 6E**). In GSE135222 database, the AUC scores of duplets, BAG3^+^IFITM2^+^ CAFs, and BAG3^+^CD8^+^ T cells were 0.89, 0.83, and 0.74, respectively (**Figure 6F**).

Compared with BAG3^+^IFITM2^+^ CAFs and BAG3^+^CD8^+^ T cells, BAG3^+^ CAF-T Cell Neighborhood exhibited the best predictive performance. Therefore, we analyzed the level of the BAG3^+^ CAF-T Cell Neighborhood, as well as its impact on survival, in multiple public databases. In NSCLC-GSE126044, centralized signature score of duplets in the non-response group is significantly higher than that in the response group (*p*=0.031). In NSCLC-GSE135222, normalized signature score of duplets in the non-response group is significantly higher than that in the response group (*p*=0.043) (**Figure 6G**). In NSCLC-37024582, the OS of the duplets high group is significantly decreased than that of the low group (*p*=0.021) (**Figure 6H**). The PFS of the duplets low group is significantly better than that of the high group (*p*=0.0015) (**Figure 6I**). In NSCLC-GSE136961, OS was significantly poorer in the duplets high group than in the low group (*p*=0.0012) (**Figure 6J**). Also, the PFS of the duplets high group is significantly shorter than that of the low group in NSCLC-GSE93157 (*p*=0.024) (**Figure 6K**). These results indicate that BAG3^+^ CAF-T Cells Neighborhood is an effective biomarker for predicting non-response to NCIT treatment.

## 4 Discussion

In this study, we found that the level of BAG3^+^ CAF-T Cells Neighborhood outperformed BAG3^+^IFITM2^+^ CAFs and BAG3^+^CD8^+^ T cells in predicting NCIT treatment outcomes. Furthermore, high levels of BAG3^+^ CAF-T Cell Neighborhoods were strongly associated with decreased DFS and OS in NSCLC patients treated with NCIT. These findings highlight the critical role of the BAG3^+^ CAF-T Cell Neighborhood in NCIT response and underscore their potential as a reliable predictor of non-response to NCIT.

The identification of PD-L1 as an immune checkpoint has revolutionized treatment paradigms for NSCLC (30). While NCIT has demonstrated superior OS benefits compared to chemotherapy alone in resectable NSCLC, approximately 40% of patients still exhibit no response to NCIT and fail to achieve MPR (31, 32). In this study, our analysis demonstrated significantly improved OS and DFS in patients achieving MPR compared to nMPR, underscoring the critical importance of attaining MPR through NCIT for optimizing outcomes in resectable NSCLC. While our data suggested a trend toward improved DFS and OS with higher PD-L1 expression, this association did not reach statistical significance, consistent with previous reports that question PD-L1’s reliability as a standalone predictive biomarker for NCIT response (33, 34). These findings highlight an urgent need to identify novel biomarkers that can predict NCIT non-response and adjust the treatment strategy.

Current radiological assessment was demonstrated limited predictive value for nMPR following NCIT, as established in previous studies (35). Our clinical data revealed a notable discrepancy between radiological and pathological responses. These findings highlight the potential limitations of relying solely on traditional radiological-based assessments to evaluate treatment responses, which may lead to inaccurate evaluations of patients’ treatment outcomes. This discrepancy may be attributed to the mechanism of action of immunotherapy, which induces distinct response patterns mediated by T cells, CAFs, and other cells in the TME. The treatments often modify the cellular activity and status within the TME, limiting the accuracy of radiological-based assessments in clinical benefits. This significant false-positive rate (40.35%) underscores both the clinical imperative to identify more reliable predictors of MPR and the critical need for therapeutic intensification strategies in this discordant population (PR-nMPR). Targeted interventions combining NCIT with additional treatment modalities in this subgroup may represent a promising approach to improve pathological response rates and, consequently, long-term patient outcomes.

Our analysis revealed significant activation of stress-related pathways from non-responders, with particularly prominent activity in CAFs and T cells. Analysis demonstrated marked upregulation of multiple stress-response markers in nMPR cases, most notably BAG3 and IFITM2. CAFs exhibit remarkable functional heterogeneity (36). This CAF heterogeneity directly influences TME, thereby offering potential targets for personalized therapeutic strategies. CD8^+^ T cells are important cells involved in regulating the efficacy of immunotherapy for NSCLC. The CD8^+^ nTIL (tumor-infiltrating lymphocytes in necrotic areas) density is a robust predictor of EFS in NSCLC patients treated with neoadjuvant immunochemotherapy (37). Notably, apCAFs demonstrate significant enrichment in non-MPR TME and mediate CD8^+^ T cell suppression via the PD-L2-RGMB axis (12). Previous studies have demonstrated that USP32 promotes NSCLC progression through BAG3 deubiquitination (38). While IFITM2 has been shown to drive gastric cancer growth and metastasis (39).

Furthermore, our data demonstrated significant enrichment of BAG3^+^IFITM2^+^ CAFs and BAG3^+^CD8^+^ T cells in nMPR patients. Amanda L. identified the PD1-PD-L1 interaction as a functional predictor for response to ICI therapy in NSCLC (40). The BAG3^+^IFITM2^+^ CAFs and BAG3^+^CD8^+^ T cells are spatially adjacent to each other, so we define this structure as BAG3^+^ CAF-T Cell Neighborhood. CAF phenotypes and CD8^+^ T cells are associated with patients’ outcome in NSCLC (20). We evaluated the predictive performance of BAG3^+^IFITM2^+^ CAFs, BAG3^+^CD8^+^ T cells, and BAG3^+^ CAF-T Cell Neighborhood for non-response to NCIT respectively, and found that BAG3^+^ CAF-T Cell Neighborhood exhibited the best performance. This may be due to the fact that it integrates two cell types, CAFs and CD8^+^ T cells, for prediction. These results suggest that targeted modulation of BAG3-based CAF-T Cell Neighborhood could potentially improving clinical outcomes.

A limitation of the public dataset validation is that most available NSCLC immunotherapy transcriptomic cohorts were generated from advanced-stage patients treated with anti-PD-1/PD-L1 therapy outside the neoadjuvant setting. Their endpoints were mainly RECIST-defined response, durable clinical benefit, PFS or OS, rather than pathological MPR after NCIT. Moreover, bulk RNA-seq and nCounter datasets do not preserve spatial coordinates and therefore cannot directly quantify the mIF-defined BAG3^+^ CAF-T Cell Neighborhood. These analyses should therefore be interpreted as external transcriptomic support for a BAG3-related CAF–CD8 T-cell resistance program, while prospective validation in independent pre-treatment NCIT cohorts with spatially resolved profiling will be required to establish its NCIT-specific predictive utility.

To successfully translate the BAG3^+^ CAF-T Cell Neighborhood from a benchside discovery into a bedside diagnostic tool, several practical implementation considerations must be delineated.

First, regarding diagnostic feasibility, neoadjuvant treatment decisions are exclusively reliant on small pre-treatment core needle biopsies (CNBs). While multiplex immunofluorescence (mIF) is highly compatible with routine FFPE specimens, the inherent spatial heterogeneity of the tumor microenvironment raises concerns about sampling bias. To ensure reproducible measurement, future clinical protocols must establish strict pre-analytical quality control metrics, such as defining a minimum requisite viable tumor area or mandating the evaluation of multiple independent spatial fields (FOVs) per biopsy core.

Second, the proposed spatial score cut-off holds significant potential for risk stratification. If a patient’s pre-treatment biopsy exhibits a high density of BAG3^+^ CAF-T Cell Neighborhoods (classifying them as at high risk for NCIT non-response), clinicians could proactively deviate from standard chemoimmunotherapy to avoid unnecessary toxicities. Such ‘high-risk’ patients might be prioritized for intensified therapeutic strategies, such as the incorporation of novel CAF-targeting agents, localized radiotherapy, or direct surgical resection. Conversely, ‘low-risk’ patients could confidently proceed with standard NCIT regimens.

Finally, realizing this clinical implementation pathway necessitates robust prospective validation. The current retrospective evaluation must be succeeded by the development of automated, artificial intelligence (AI)-assisted digital pathology pipelines to standardize spatial neighborhood scoring within CLIA-certified environments. Most importantly, biomarker-driven, multi-center prospective clinical trials are imperative to definitively validate its clinical utility.

We acknowledge several limitations in our study. First, the sample size was relatively small; thus, validation in a larger, multicenter cohort is required to enhance the reliability of our findings. Second, we selected a cutoff value of 2 for the BAG3^+^ CAF-T Cell Neighborhood based on the Youden index, which yielded a robust predictive model, yet we did not explore other potential thresholds. Third, our results suggest that the BAG3-IFITM2 axis exerts an inhibitory effect on CD8^+^ T cell function, but the precise mechanistic role of BAG3^+^IFITM2^+^ CAFs, particularly their interaction with BAG3^+^CD8^+^ T cells, remains to be fully elucidated and warrants further experimental investigation.

## 5 Conclusions

In conclusion, our study establishes the first benchmark for characterizing BAG3^+^ CAF-T Cell Neighborhood in response to NCIT in NSCLC patients. We demonstrate that the level of BAG3^+^ CAF-T Cell Neighborhood, as quantified by mIF, represents a clinically feasible and reproducible method for predicting NCIT treatment outcomes. Thus, the level of BAG3^+^ CAF-T Cell Neighborhood holds promise as a predictor of non-response in NCIT. Consequently, our findings demonstrate the necessity for combined diagnostic approaches and therapeutic strategies. Therapeutic targeting of BAG3^+^IFITM2^+^ CAFs and BAG^+^CD8^+^ T cells represents a promising adjunct to enhance NCIT efficacy in NSCLC treatment.

## Data Availability

The data that support the findings of this study are openly available in public repositories. The single-cell RNA sequencing data have been deposited in the GEO repository, accession number GSE337519.
