# Supplementary material for: BAG3+ CAF-T cell neighborhood predicts resistance to neoadjuvant chemoimmunotherapy in NSCLC
Source: Front Immunol. 2026 Jul 7;17:1823199. doi: 10.3389/fimmu.2026.1823199 (PMC13385752; doi:10.3389/fimmu.2026.1823199)
Supplement: Supplementary file 1 [file Supplementaryfile1.pdf]

Supplementary data

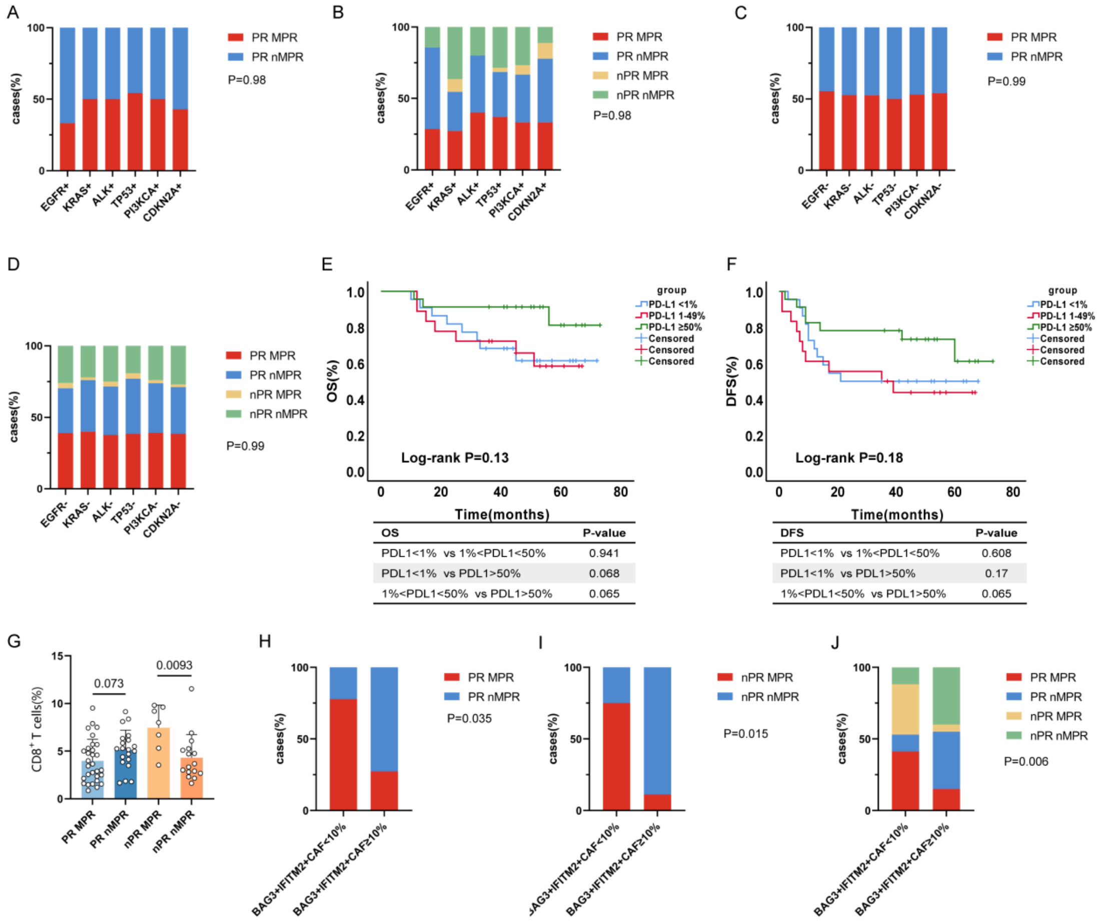

FIGURE S1

Comparative analysis of different groups. **(A)** The proportions of EGFR+, KRAS+, ALK+, TP53+, PI3KCA+, CDKN2A+ cases in PR-MPR and PR-nMPR groups. **(B)** The proportions of EGFR+, KRAS+, ALK+, TP53+, PI3KCA+, CDKN2A+ cases among PR-MPR, PR-nMPR, nPR-MPR and nPR-nMPR groups. **(C)** The proportions of EGFR-, KRAS-, ALK-, TP53-, PI3KCA-, CDKN2A- cases in PR-MPR and PR-nMPR groups. **(D)** The proportions of EGFR-, KRAS-, ALK-, TP53-, PI3KCA-, CDKN2A- cases among PR-MPR, PR-nMPR, nPR-MPR and nPR-nMPR groups. **(E)** Kaplan–Meier curves analysis of OS among the PDL1< 1%, PDL1 1–49% and PDL1 ≥ 50% groups. **(F)** Kaplan–Meier curves analysis of DFS among the PDL1< 1%, PDL1 1–49% and PDL1 ≥ 50% groups. **(G)** Column charts showing the proportions of $CD8^+$ T cells in PR-MPR, PR-nMPR, nPR-MPR and nPR-nMPR groups. P value is calculated using unpaired Student's t test. **(H)** The proportions of different $BAG3^+IFITM2^+$ CAFs expression levels in PR-MPR and PR-nMPR groups. **(I)** The proportions of different $BAG3^+IFITM2^+$ CAFs expression levels in the nPR-MPR and nPR-nMPR groups. **(J)** The proportions of different $BAG3^+IFITM2^+$ CAFs expression levels among PR-MPR, PR-nMPR, nPR-MPR and nPR-nMPR groups.

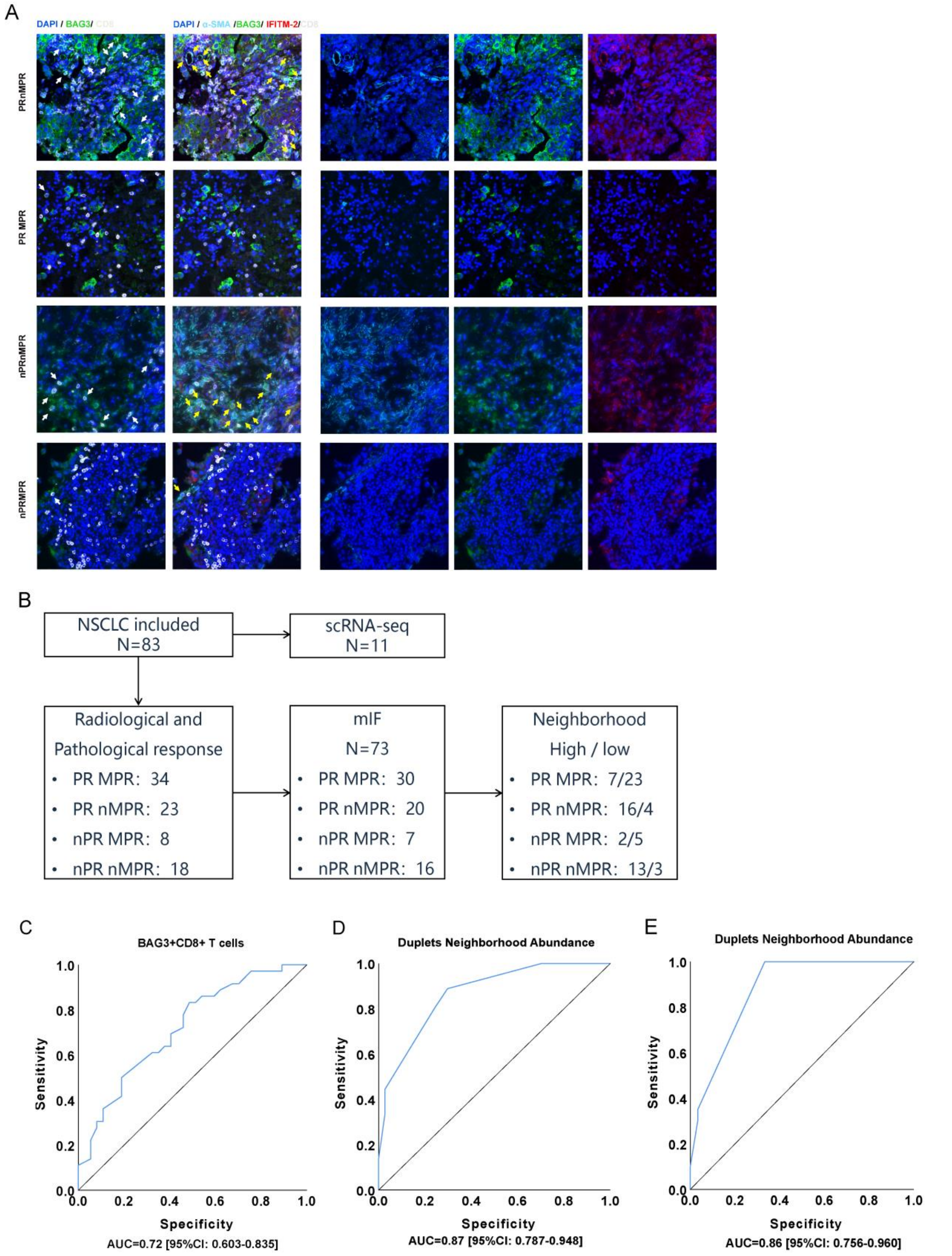

FIGURE S2

BAG3$^+$IFITM2$^+$ CAFs and BAG3$^+$CD8$^+$ T cells are spatially adjacent. **(A)** The yellow arrows indicate the spatial distribution of BAG3$^+$IFITM2$^+$ CAFs. The white arrows indicate the spatial distribution of BAG3$^+$CD8$^+$ T cells. **(B)** The patient-flow diagram. **(C)** ROC curve of BAG3$^+$CD8$^+$ T cells; AUC and 95% CI, n=73. **(D)** ROC curve of BAG3$^+$ CAF-T Cells Neighborhood; AUC and 95% CI, n=73. **(E)** ROC curve of BAG3$^+$ CAF-T Cells Neighborhood in PR patients; AUC and 95% CI.
